# The Off-Label Use of a Lumen-Apposing Metal Stent (LAMS) for a Benign Colon Anastomotic Stricture Causing Recurrent Bowel Obstruction in a Patient with Keloids

**DOI:** 10.1155/2021/5595518

**Published:** 2021-04-02

**Authors:** Michael Coles, Pearl Uy, Victoria Madray, John Erikson Yap, Subbaramiah Sridhar

**Affiliations:** ^1^Augusta University, Department of Internal Medicine, 1120 15th Street, Augusta, Georgia, USA; ^2^Augusta University, Department of Gastroenterology and Hepatology, 1120 15th Street, Augusta, Georgia, USA; ^3^Augusta University, Medical College of Georgia, 1120 15th Street, Augusta, Georgia, USA

## Abstract

Anastomotic strictures are a known complication of colorectal surgery. Despite a wide range of medical devices that have been deployed for this complication, outcomes remain challenging. Lumen-apposing metal stents (LAMSs) have recently emerged as a potentially superior therapeutic option. We herein report a patient with a past medical history of pT3, N0 adenocarcinoma of the colon with anastomotic stricture recurrence who underwent successful placement of an LAMS. We suggest that patients with a predisposition for keloid formation or fibrosis-prone anastomotic wound healing should be considered for LAMS deployment early in the treatment course.

## 1. Introduction

Benign colon anastomotic strictures can lead to recurrent hospitalizations from bowel obstruction. Most benign gastrointestinal (GI) strictures are managed endoscopically with balloon dilation, intralesional steroid injection, incisional therapy, and/or self-expanding metal stents (SEMSs) [1]. However, strictures affecting the GI tract can be recalcitrant. We present the successful treatment of a benign recurrent sigmoid anastomotic stricture with a Lumen-Apposing Metal Stent (LAMS).

## 2. Case Report

We present a 67-year-old African-American male patient who underwent sigmoidectomy, diverting ostomy, and then, subsequent reversal under adjuvant chemotherapy for a pT3, N0 adenocarcinoma of the colon. His ostomy was complicated by recurrent keloid formation causing bowel obstruction prior to the ostomy reversal. He presented to the emergency department with abdominal pain, nausea, and constipation of two-week duration. He had tried multiple over-the-counter laxatives, which failed to abate his symptoms. He was not receiving opioid therapy.

**Figure 1:**
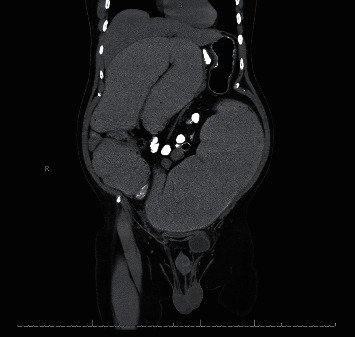
Coronal section of computed tomography without contrast of the abdomen and pelvis demonstrating significant dilation of the colon proximal to the colonic anastomosis from prior sigmoidectomy consistent with an obstructing anastomotic stricture.

**Figure 2:**
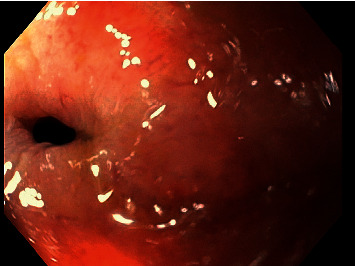
Flexible sigmoidoscopy 20 cm from the anal verge demonstrating a 10 mm-long anastomotic stricture with a luminal diameter of 8 mm.

Upon presentation, the patient's vital signs were normal. On physical examination, the patient had a significantly distended, nontender abdomen and hyperactive bowel sounds with tympany. His laboratory parameters were unremarkable. The abdominal X-ray demonstrated a dilated colon, and a follow-up CT scan demonstrated significant dilation of the colon measuring approximately 20 cm in diameter just proximal to the colonic anastomosis concerning for an obstructing anastomotic stricture **(**Figure 1**)**. A flexible sigmoidoscopy revealed a 10 mm-long anastomotic stricture at 20 cm from the anal verge **(**Figure 2**)** with a luminal diameter of 8 mm. The stricture was subsequently dilated over multiple endoscopic sessions in a stepwise manner to attain a maximal stricture luminal diameter of 20 mm. However, over the following 6 months, the anastomotic stricture recurred, causing colonic obstruction requiring at least 4 more dilatation sessions.

**Figure 3:**
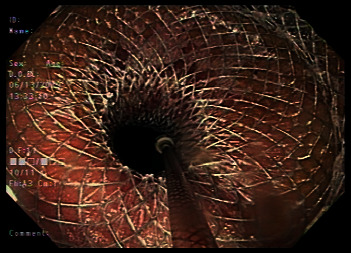
Retroflexion perspective demonstrating the proximal aspect of the deployed LAMS.

Given the recalcitrant nature of the patient's anastomotic stricture, he was offered alternative therapies including placement of a Lumen-Apposing Metal Stent (LAMS) or surgical resection of the stricture. Due to the history of keloid formation, the patient elected to undergo LAMS placement. A flexible sigmoidoscopy was performed, and a 20 mm × 10 mm LAMS was successfully deployed at the anastomotic stricture site. The proximal aspect of the deployed LAMS was inspected with retroflexion with an ultrathin upper endoscope to ensure appropriate positioning of the stent **(**Figure 3).

**Figure 4:**
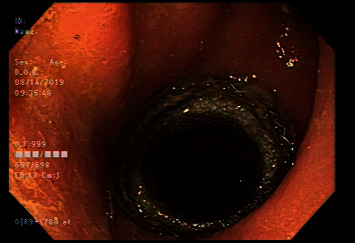
Flexible sigmoidoscopy illustrating a patent LAMS in place over an anastomotic stricture.

The patient did well for 6 months following the stent placement. Subsequent flexible sigmoidoscopy showed the stent to be in good position with maintained patency (Figure 4). He had no abdominal pain, constipation, or rectal bleeding. He reported one normal bowel movement every 2-3 days while on a laxative. A flexible sigmoidoscopy with removal of the LAMS was performed with notable clean base ulcerations at the previous location of the stent. This was followed by injection of 40 mg of triamcinolone (10 mg into each of the four quadrants) into the previously strictured area. A follow-up flex sigmoidoscopy was performed after 13 months, which showed a patent anastomotic site, and repeat 4-quadrant triamcinolone injection was performed. The patient continues to remain asymptomatic without any hospitalizations for obstructive symptoms 5 months since his last flex sigmoidoscopy.

## 3. Discussion

Anastomotic strictures resulting from colorectal surgery are therapeutically challenging and often resistant to the established therapies including balloon dilations, incisional therapy, and deployment of biodegradable (BDS) or fully covered self-expanding metal stents (FC-SEMS) [1]. Lumen-apposing metal stents (LAMSs) were originally indicated to facilitate access and drainage of pancreatic fluid collections [1]. However, LAMS have recently emerged as a potentially superior clinical alternative to FC-SEMS. The therapeutic benefit of FC-SEMS may be undermined by their high migration rates and limited efficacy demonstrated in several trials [2]. The LAMS imparts lumen apposition via its wide flanges, which provides an anchoring mechanism, features lacking in FC-SEMS. FC-SEMS can be anchored by means of stent suturing but it is both expensive [3] and more technically challenging for the endoscopist, ultimately affecting the likelihood of procedural success [4]. In esophageal stenting studies with suturing of stent in-situ, migration rates still ranged as high as 33% [5] and 50% when not sutured [6]. Furthermore, stent suturing with FC-SEMS is also described to cause stricture overgrowth [3]. LAMS also are manufactured with larger lumen diameters of 10, 15, and 20 mm compared to the 8 and 10 mm of FC-SEMS, theoretically reducing the likelihood of obstruction when relatively larger stents are selected for use. In a 2019 systematic review and meta-analysis by Mohan et al., management using LAMS demonstrated statistically better outcomes, including lower rates of stent migration and postprocedure pain relative to FC-SEMS and BDS [7]. Although LAMSs are less likely to migrate, their comparatively shorter length may result in misalignment of the stent lumen relative to the stricture axis resulting in obstructive symptoms [3]. As such, careful characterization of stricture morphology must be taken into consideration prior to selection of stent type. Given the off-label use of an LAMS in benign gastrointestinal strictures, no definitive guidelines exist when selecting an optimal stent size. We opted for a conservative approach, with the smallest LAMS size of 10 mm to which our patient responded well. The decision to place an LAMS was motivated by the history of suboptimal therapeutic response to serial dilations. Its recalcitrant nature was speculated to partially be a sequelae of abnormal proliferation of keloidal tissue at the anastomotic site to which the LAMS tubular structure could facilitate a tract formation while maintaining lumen patency.

Postintervention information is limited mostly to case series with only short follow-up duration; however, the possibility of LAMS placement as a destination therapy may warrant consideration an individualized basis. Reintervention rates of 75% were noted at 300 days after LAMS removal in one study [8], and given such a high rate, LAMSs placed indefinitely is a therapeutic option. While placement could obviate the need for further intervention, patients may be at increased risk for bleeding, migration, stent burial or occlusion [9], and infection. Future prospective controlled trials are required to determine the long-term efficacy and risks associated with such a strategy.

In summary, this case highlights the use of an LAMS as an effective approach in the management of refractory colonic stricture. This device features ease of deployment and favorable safety profile, which yields effective palliation, recommending it as an excellent treatment option for recurrent bowel obstruction associated with a refractory benign colonic stricture. Less than 20 cases have been reported in the literature regarding the off-label use of an LAMS in the treatment of colonic strictures [10]. The accrual of larger patient experience will be important to evaluate the long-term efficacy of LAMSs in specific disease indications. Drug-elution capability allowing locoregional drug delivery addressing a wide range of benign and malignant lesions represents a potential future design modification that may enhance the utility of this minimally invasive treatment approach. Interestingly, there are multiple instances in the literature of gastrointestinal keloidal manifestations with subsequent motility complications [11–13]. Predisposition for keloid formation and altered anastomotic wound healing in patients with a benign stricture may indicate early implementation of LAMSs to achieve effective and facile restoration of colonic patency.

## Data Availability

No data were used to support this study.
